# Preoperative Significance of Ipsilateral Manual Neck Compression in Patients With Pulsatile Tinnitus Secondary to Sigmoid Sinus Dehiscences and Diverticula

**DOI:** 10.3389/fneur.2022.869244

**Published:** 2022-03-17

**Authors:** Seung Jae Lee, Sang-Yeon Lee, Byung Yoon Choi, Ja-Won Koo, Sung Hwa Hong, Jae-Jin Song

**Affiliations:** ^1^Department of Otorhinolaryngology-Head and Neck Surgery, Seoul National University Bundang Hospital, Seongnam, South Korea; ^2^Department of Otorhinolaryngology-Head and Neck Surgery, Seoul National University Hospital, Seoul, South Korea; ^3^Department of Otorhinolaryngology-Head and Neck Surgery, Seoul National University College of Medicine, Seoul, South Korea; ^4^Sensory Organ Research Institute, Seoul National University Medical Research Center, Seoul, South Korea; ^5^Department of Otorhinolaryngology-Head and Neck Surgery, Samsung Changwon Hospital, Sungkyunkwan University School of Medicine, Changwon, South Korea

**Keywords:** tinnitus, pulsatile tinnitus, pure-tone audiometry, hearing loss, jugular veins, compression

## Abstract

Venous pulsatile tinnitus (PT) is characterized by an auditory perception of pulse-synchronous sound, suppressed by compression of the ipsilateral internal jugular vein. We sought to determine the preoperative prognostic significance of the effect of ipsilateral neck manual compression on the PT loudness and audiometric changes in patients with sigmoid sinus dehiscences (SS-Deh) and diverticula (SS-Div) by comparing postoperative improvements in ipsilateral low-frequency hearing loss (LFHL) in pure-tone audiogram (PTA) and PT symptoms. Twenty-two subjects with PT originating from SS-Deh/Div were recruited. Air-conduction hearing thresholds were measured using PTA at three time points: twice preoperatively (with neutral neck position and with ipsilateral manual compression of internal jugular vein) and once at 3-months postoperatively with neutral neck position. We defined a positive neck compression effect as a threshold improvement of ≥ 10 dB HL at 250 or 500 Hz after manual neck compression. All but two subjects presented with ipsilateral LFHL in the neutral position. The average hearing threshold in the neutral position markedly improved after manual neck compression, indicating that LFHL originated from the masking effect of venous PT. All subjects had subjective improvements in PT and LFHL after sigmoid sinus surgeries, confirming that LFHL resulted from the masking effect of PT. Additionally, improvement of LFHL after neck compression could be regarded as a positive prognostic indicator after surgery. Collectively, elimination of PT loudness and improvement of LFHL with manual compression over the ipsilateral neck may suggest the venous origin of the PT and predict a favorable outcome following repair of SS-Deh/SS-Div.

## Introduction

Tinnitus can be pulsatile or non-pulsatile depending on the characteristics of the perceived sound (1). Non-pulsatile tinnitus, often referred to as subjective tinnitus, is presumably caused by functional changes in the auditory and non-auditory cortices (2, 3). Meanwhile, pulsatile tinnitus (PT) is caused by altered vascular hemodynamics, such as turbulent blood flow or vibration of a dehiscent vascular wall, or abnormal perception of the sound of normal flow, such as third-window lesions due to bony defects of the inner ear. Venous PT is characterized by auditory perception of a pulse-synchronous sound, which is usually suppressed by compressing internal jugular vein on the symptomatic side (4, 5).

Detailed otoendoscopy, physical examination, and radiologic evaluation are necessary for evaluating PT and selecting appropriate surgical candidates (6–8). In particular, high- resolution imaging modalities, such as contrast-enhanced temporal bone computed tomography and angiography (TB-CTA), depict details of the vascular abnormalities that may precipitate PT with reference to the surrounding structures. Nonetheless, some abnormalities (e.g., high-riding jugular bulb with or without dehiscence) are incidentally found among patients without PT and radiologic images may be normal in patients with PT. Therefore, better objective tests are required to identify lesions causing PT and predict symptomatic improvement after treatment. Previous studies reported that sigmoid sinus dehiscence (SS-Deh) and sigmoid sinus diverticulum (SS-Div), which were the most frequent vascular abnormalities in patients with venous PT, were also found in individuals without PT (4, 9–11).

Increasing evidence suggests that objective tests, including transcanal sound recording and spectro-temporal analysis (TSR-STA) (1, 12), can objectively demonstrate pulse-synchronous patterns of vascular PT and predict the origin thereof. Furthermore, preoperative ipsilateral low-frequency hearing loss (LFHL) has been reported as an objective audiological marker of PT, which improves following treatment in most cases (13). LFHL may predict postoperative audiological improvement on pure-tone audiometry (PTA) at low frequencies including 250 or 500 Hz, suggesting normalization of the underlying abnormal vascular pathology and alleviation of PT. Therefore, we hypothesized that audiological evaluation while applying manual neck compression would circumvent the masking effect of PT, thereby detecting the causative lesion of PT and helping to select the appropriate surgical candidates.

In this study, we explored the preoperative diagnostic and prognostic values of the effect of manual compression over the ipsilateral neck on the PT loudness and audiometric changes in patients with SS-Div or SS-Deh by comparing the ipsilateral LFHL and PT pre- and post-operatively. By confirming our a priori hypothesis, we may be able to understand the pathophysiological mechanisms of SS-Div/SS-Deh-associated PT and contribute to the development of diagnostic techniques for PT.

## Materials and Methods

### Subjects

We screened PT patients who presented to Seoul National University Bundang Hospital, South Korea, between January 2019 and May 2021. This study included patients with radiologically documented SS-Div/SS-Deh and preoperative ipsilateral LFHL (pure-tone threshold ≥ 10 dB HL at 250 or 500 Hz compared to the symptom-free side) who underwent SS resurfacing or reshaping by a single surgeon (J-J.S.). Other arterial, venous or non-vascular etiologies of PT including intracranial arteriovenous fistulae and aneurysms, aberrant carotid artery, jugular bulb abnormalities or vascular neoplasms were excluded in our study (14). Twenty-two patients were included in this study and all of them underwent standard physical examinations at the outpatient clinic including thorough inspection and auscultation of head and neck area, and otoendoscopic examinations. The study was approved and patient consent was waived by the Institutional Review Board of the Clinical Research Institute (no. B-2107-699-105) and was conducted in accordance with the Declaration of Helsinki.

### Audiological Evaluations

The patients underwent PTA pre- and post-operatively. Air conduction thresholds for seven octave frequencies (250, 500, 1,000, 2,000, 4,000, and 8,000 Hz) were evaluated using PTA in a soundproof booth. Serial audiograms were performed to determine the PTA threshold at all frequencies, at 3 time points: preoperatively with neutral neck position, preoperatively with ipsilateral manual compression of the internal jugular vein, and 3 months postoperatively with neutral neck position. A recent study investigated audiometric threshold shifts from PT due to SS wall anomalies, demonstrating PT can cause an average of 6 dB, and uncommonly up to 30 dB, of low-frequency hearing threshold shift in the affected ear (15). Since there is no gold standard for the definition of ipsilesional LFHL (i.e., pseudo-LFHL, PLFHL), we have defined it as a threshold difference of ≥ 10 dB HL at 250 or 500 Hz between ipsi- and contra-lesional neutral positions which fell within the middle range based on previous literature. Positive manual neck compression effect was defined as a threshold improvement of ≥ 10 dB HL at 250 or 500 Hz after manual compression.

### Subjective Tinnitus Evaluations

At the initial visit, a structured interview about the PT characteristics was conducted. The patients completed a comprehensive questionnaire, which included the Numerical Rating Scale (NRS) and Tinnitus Handicap Inventory (THI) (16), to assess subjective tinnitus loudness and severity pre- and post-operatively. Pre- and post-operative NRS and THI scores were obtained at 1 day before and after surgery. The NRS consisted of 2 elements designed to assess the degree of annoyance (NRS-A; range, 0–10) and subjective loudness of tinnitus (NRS-L; range, 0–10).

### Radiologic Evaluations

The patients underwent contrast-enhanced TB-CTA using Philips 128 CT scanners (Philips Medical Systems, Eindhoven, The Netherlands). TB-CTA was performed using tube energy of 120 kVp, a quality reference value of 250 mA, and a detector configuration of 2 × 0.625 mm. The CT scan slices were of 0.7 mm and the scan length was 16 cm. Additionally, contrast-enhanced brain magnetic resonance imaging (MRI) with magnetic resonance angiography (MRA) were performed to identify pathognomonic findings related to dural arteriovenous fistula, pseudotumor cerebri, and idiopathic intracranial hypertension. Specifically, we examined for transverse sinus stenosis and empty sella, which are radiological biomarkers of diseases associated with PT (4). A neuroradiologist and an otolaryngologist (S.J.L), who were blinded to clinical findings, independently reviewed the images of brain neurovascular structures and cervical neck to detect abnormal findings associated with PT.

### Surgical Intervention

A typical cortical mastoidectomy was performed using the retro-auricular approach. As for the SS-Div cases, the diverticulum was completely skeletonized and manually reduced to the level of the adjacent SS wall, and the bony dehiscence surrounding the diverticulum was reconstructed by extraluminal placement of temporalis fascia and Mimix™ hydroxyapatite bone cement (Walter Lorenz Surgical Inc., Jacksonville, FL, USA). Meanwhile, for the SS-Deh cases, the SS and its dehiscent or thinned portions were also skeletonized for reshaping of the SS. The dominant SS was gently decompressed using autologous materials, and the SS-Deh was carefully reconstructed using temporalis fascia and bone cement as described in previous literature of ours (11, 17, 18).

### Statistical Analysis

Data are presented as mean ± standard deviation (SD). Statistical analyses were performed using SPSS software (version 20; IBM Corp., Armonk, NY, USA). The statistical results were depicted using the GraphPad Prism software (version 9.0.0; GraphPad Software Inc., San Diego, CA, USA). The hearing thresholds were pair-wise compared at each frequency between the ipsilateral and contralateral sides, between ipsilateral neutral and manual compression positions, and between pre- and post-operative neutral positions using Wilcoxon signed-rank test. Pre- and post-operative subjective symptoms in terms of NRS and THI scores were compared using the aforementioned methods. *P*-value < 0.05 was considered statistically significant.

## Results

### Demographic Characteristics

**Figure 1:**
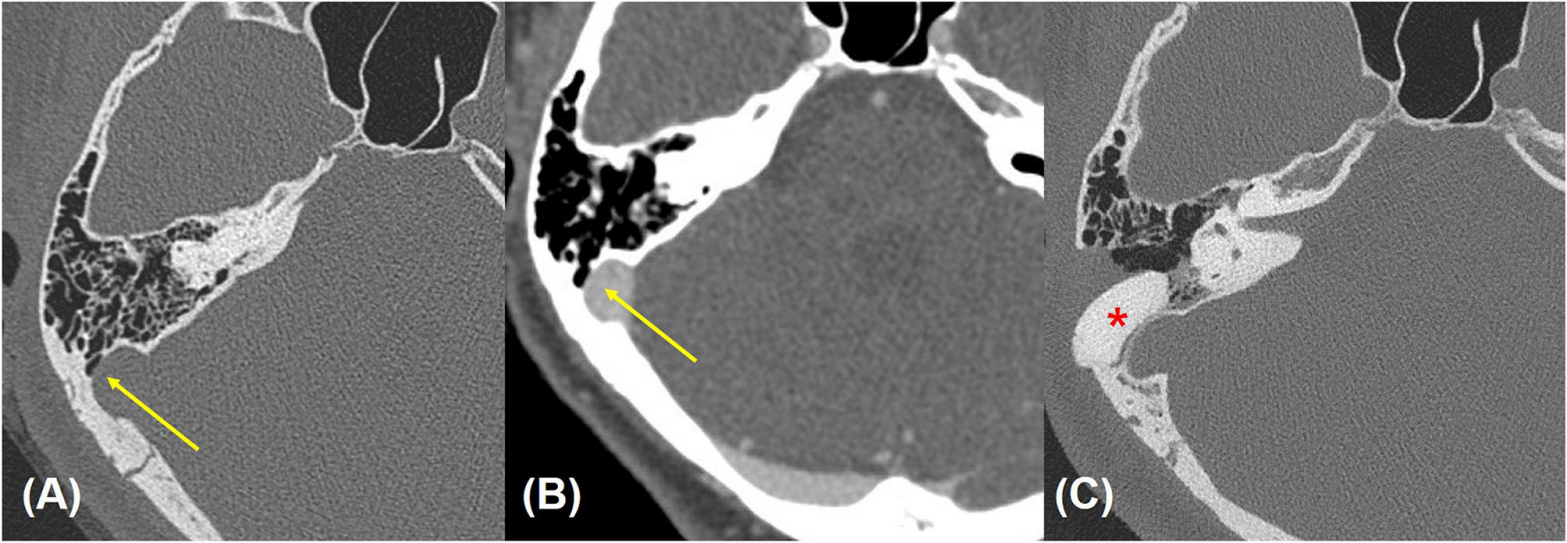
Preoperative **(A,B)** and postoperative **(C)** temporal bone computed tomographic angiography (TB-CTA) images of Subject 4. Focal bony dehiscence at the lateral wall of the right transverse-sigmoid junction is observed [yellow arrow points to precontrast **(A)** and contrast-enhanced **(B)** images]. **(C)** Postoperative TB-CTA image showing calcium hydroxyapatite well *in situ* at the previously noted defect site (red asterisk).

Table 1 presents the demographic and clinical characteristics of the 22 subjects (20 women and two men). The mean age of subjects was 33.1 ± 6.6 years (range, 19–45 years). Sixteen subjects (72.7 %) presented with right-sided PT and six (27.3 %) presented with left-sided PT. The mean duration of PT symptoms was 30.1 ± 17.3 months (range, 2–120 months). SS-Deh and SS-Div were observed on TB-CTA in 14 (63.6 %) and 8 (36.4 %) subjects, respectively. Fifteen subjects (80.0 %) underwent SS operations; six of them were diagnosed with SS-Div and underwent SS resurfacing, while nine underwent SS reshaping due to SS-Deh. Postoperatively, SS and its dehiscence or diverticulum were found to be completely corrected in all subjects after reviewing TB-CTA taken 3 months after surgical interventions. A representative case of SS-Deh is demonstrated in Figure 1.

**Table 1:** Demographic and clinical characteristics of our cohort.

**Pt. No**.	**Sex**	**Age**	**Side**	**Sx.Duration (mo)**	**Diagnosis**	**Operation name**
1	F	24	R	48	(R) SS diverticulum	(R) SS resurfacing
2	F	42	R	9	(R) SS dehiscence	(R) SS reshaping
3	F	32	L	60	(L) SS dehiscence	(L) SS reshaping
4	F	30	R	4	(R) SS dehiscence	(R) SS reshaping
5	F	42	R	3	(R) SS diverticulum	(R) SS resurfacing
6	F	27	R	13	(R) SS dehiscence	(R) SS reshaping scheduled
7	F	45	R	24	(R) SS dehiscence	(R) SS reshaping
8	M	29	R	12	(R) SS dehiscence	(R) SS reshaping
9	F	25	R	10	(R) SS dehiscence	(R) SS reshaping
10	F	40	R	36	(R) SS dehiscence	None
11	F	28	R	24	(R) SS diverticulum	(R) SS resurfacing
12	F	36	L	9	(L) SS dehiscence	(L) SS reshaping
13	F	26	R	36	(R) SS dehiscence	(R) SS reshaping
14	F	33	R	2	(R) SS dehiscence	(R) SS reshaping
15	F	37	R	4	(R) SS diverticulum	(R) SS resurfacing
16	F	41	L	48	(L) SS diverticulum	(L) SS resurfacing scheduled
17	M	45	R	120	(R) SS diverticulum	(R) SS resurfacing scheduled
18	F	19	R	12	(R) SS dehiscence	None
19	F	36	L	84	(L) SS diverticulum	(L) SS resurfacing
20	F	23	L	8	(L) SS dehiscence	None
21	F	26	R	48	(R) SS dehiscence	(R) SS reshaping scheduled
22	F	20	L	48	(L) SS diverticulum	(L) SS resurfacing

*Pt. No., patient number; M, male; F, female; R, right; L, left; Sx., symptom; mo, month; SS, sigmoid sinus*.

*Of 22 subjects, 20 (90.9%) were women. The mean age of subjects was 33.1 ± 6.6 years (range, 19–45 years). Sixteen subjects (72.7 %) presented with right-sided pulsatile tinnitus (PT) and six (27.3 %) with left-sided PT. The mean duration of PT symptoms was 30.1 ± 17.3 months (range, 2–120 months). According to temporal bone computed tomography and angiography (TB-CTA), 14 (63.6%) and 8 (36.4%) showed SS-Deh and SS-Div, respectively. Of the 15 subjects who underwent surgery, 6 (40.0%) and 9 (60.0%) underwent SS resurfacing due to SS-Div and SS reshaping due to SS-Deh, respectively*.

### Effect of Manual Neck Compression on Audiological Manifestation

The pre- and post-operative audiological profiles of both 250 and 500 Hz are summarized in Table 2. In the neutral position, all but two subjects (Subjects 13 and 20, 90.9%) had ipsilateral LFHL compared to the contralateral side. The average hearing thresholds of neutral position at both 250 and 500 Hz were significantly different between the ipsilateral and contralateral sides (27.5 ± 8.2 dB HL and 8.9 ± 4.5 dB HL at 250 Hz, 20.2 ± 7.9 dB HL and 9.1 ± 4.4 dB HL at 500 Hz, respectively; both *p* < 0.001, the Wilcoxon signed-rank test). Sixteen subjects (72.7 %) had a positive ipsilateral effect of manual compression (i.e., improvement of ≥ 10 dB HL at 250 or 500 Hz). The average hearing threshold markedly improved from 27.5 ± 8.2 dB HL to 14.3 ± 6.3 dB HL at 250 Hz and from 20.2 ± 8.0 dB HL to 12.1 ± 5.6 dB HL at 500 Hz (both *p* < 0.001, the Wilcoxon signed-rank test) after manual neck compression, indicating that the LFHL was a result of the masking effect of venous PT. However, manual compression did not elicit a significant difference in hearing thresholds at other frequencies (1,000, 2,000, 4,000, or 8,000 Hz) compared to the neutral position.

**Table 2:** Effect of ipsilesional neck compression on low-frequency hearing thresholds.

**Pt. No**.	**250 Hz**			**500 Hz**			**PLFHL**	**Ipsilesional neck compression effect**
	**Ipsi-lesional neutral (dB HL)**	**Contra-lateral neutral (dB HL)**	**Ipsi-lesional neck com. (dB HL)**	**Ipsi-lesional neutral (dB HL)**	**Contra-lateral neutral (dB HL)**	**Ipsi-lesional neck com. (dB HL)**		
1	20	5	5	15	5	5	O	O
2	35	5	10	20	10	15	O	O
3	30	10	15	30	10	20	O	O
4	30	5	15	15	10	15	O	O
5	20	10	20	10	5	10	O	X
6	20	10	15	20	10	15	O	X
7	20	10	15	5	5	10	O	X
8	35	5	10	20	0	5	O	O
9	35	20	15	20	15	10	O	O
10	20	5	20	15	5	20	O	X
11	15	5	10	15	10	10	O	X
12	20	5	5	15	5	5	O	O
13	25	20	20	20	20	10	X	O
14	20	10	10	20	10	10	O	O
15	25	10	15	25	10	10	O	O
16	40	10	10	30	10	5	O	O
17	35	5	10	20	5	15	O	O
18	30	10	15	30	10	10	O	O
19	45	5	25	40	10	25	O	O
20	20	15	20	20	15	15	X	X
21	25	5	5	10	5	5	O	O
22	40	10	30	30	15	20	O	O

*Pt. No., patient number; PTA, pure tone audiogram; PLFHL, pseudo-low-frequency hearing loss; com., compression*.

*In the neutral position, all but two subjects (Subjects 13 and 20) had ipsilateral low-frequency hearing loss (LFHL) (i.e., pseudo-low-frequency hearing loss, PLFHL) compared to the contralateral side. Sixteen subjects (72.7 %) had a positive ipsilateral effect of manual neck compression (i.e., improvement of ≥ 10 dB HL at 250 or 500 Hz), as proposed by our study. In the neutral position, the average hearing thresholds at 250 and 500 Hz were significantly higher in the ipsilateral side than the contralateral side (27.5 ± 8.2 dB HL vs. 8.9 ± 4.5 dB HL at 250 Hz, 20.2 ± 7.9 dB HL vs. 9.1 ± 4.4 dB HL at 500 Hz, respectively; both p < 0.001). After manual neck compression, the average hearing threshold markedly improved from 27.5 ± 8.2 dB HL to 14.3 ± 6.3 dB HL at 250 Hz and from 20.2 ± 8.0 dB HL to 12.1 ± 5.6 dB HL at 500 Hz (both p < 0.001)*.

### Pre- and Postoperative Audiological Manifestations

**Figure 2:**
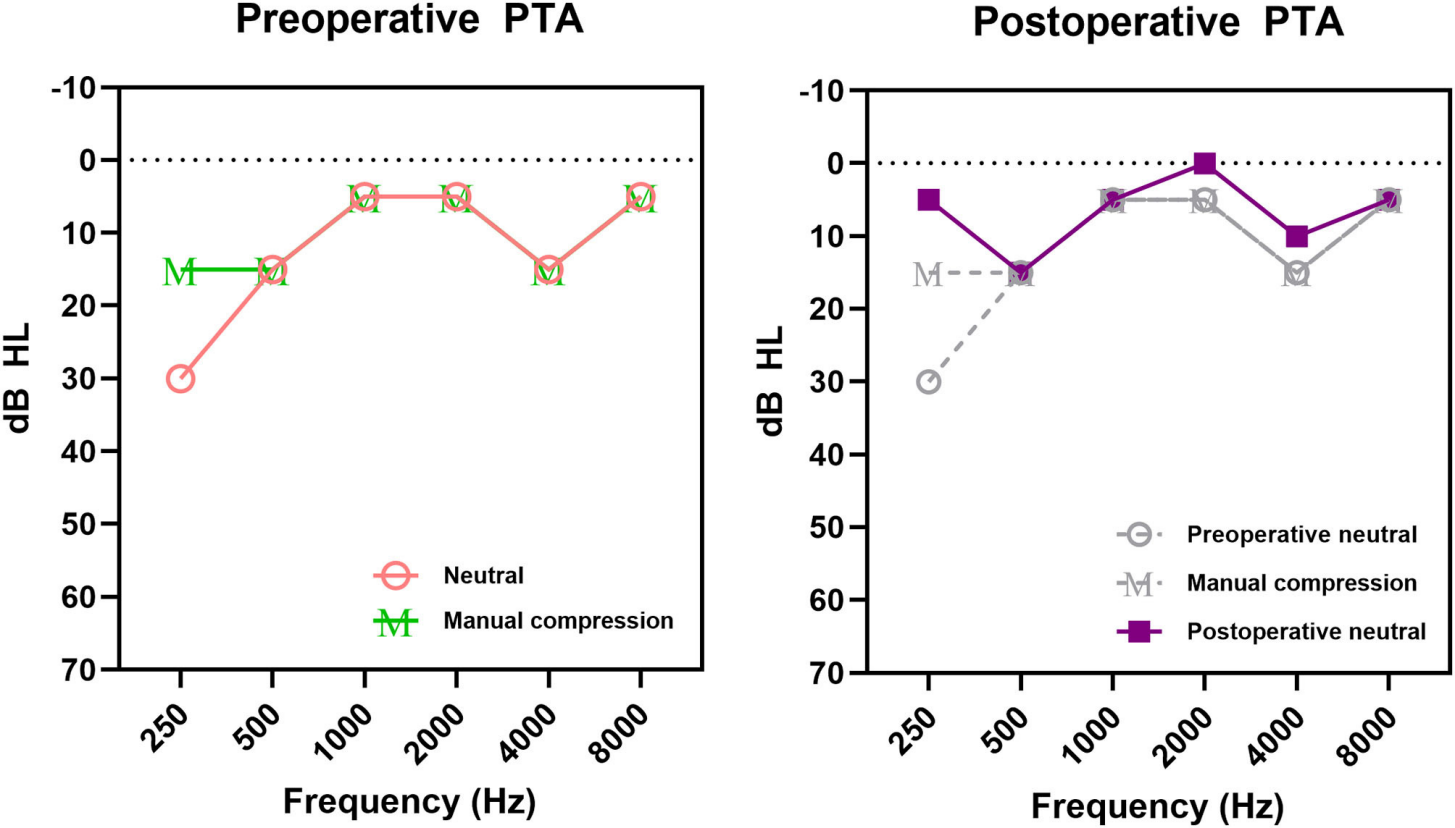
Pure-tone audiogram (PTA) of Subject 4 with pulsatile tinnitus and ipsilateral low-frequency hearing loss (LFHL). Ipsilateral LFHL was defined as a hearing threshold >10 dB HL at 250 or 500 Hz compared to the opposite symptom-free side; positive neck compression effect was defined as a threshold decrease of ≥ 10 dB HL at 250 or 500 Hz after manual compression. Changes in ipsilateral pure-tone thresholds after neck compression and surgical treatment are demonstrated. M in preoperative PTA represents hearing thresholds after manual compression.

**Figure 3:**
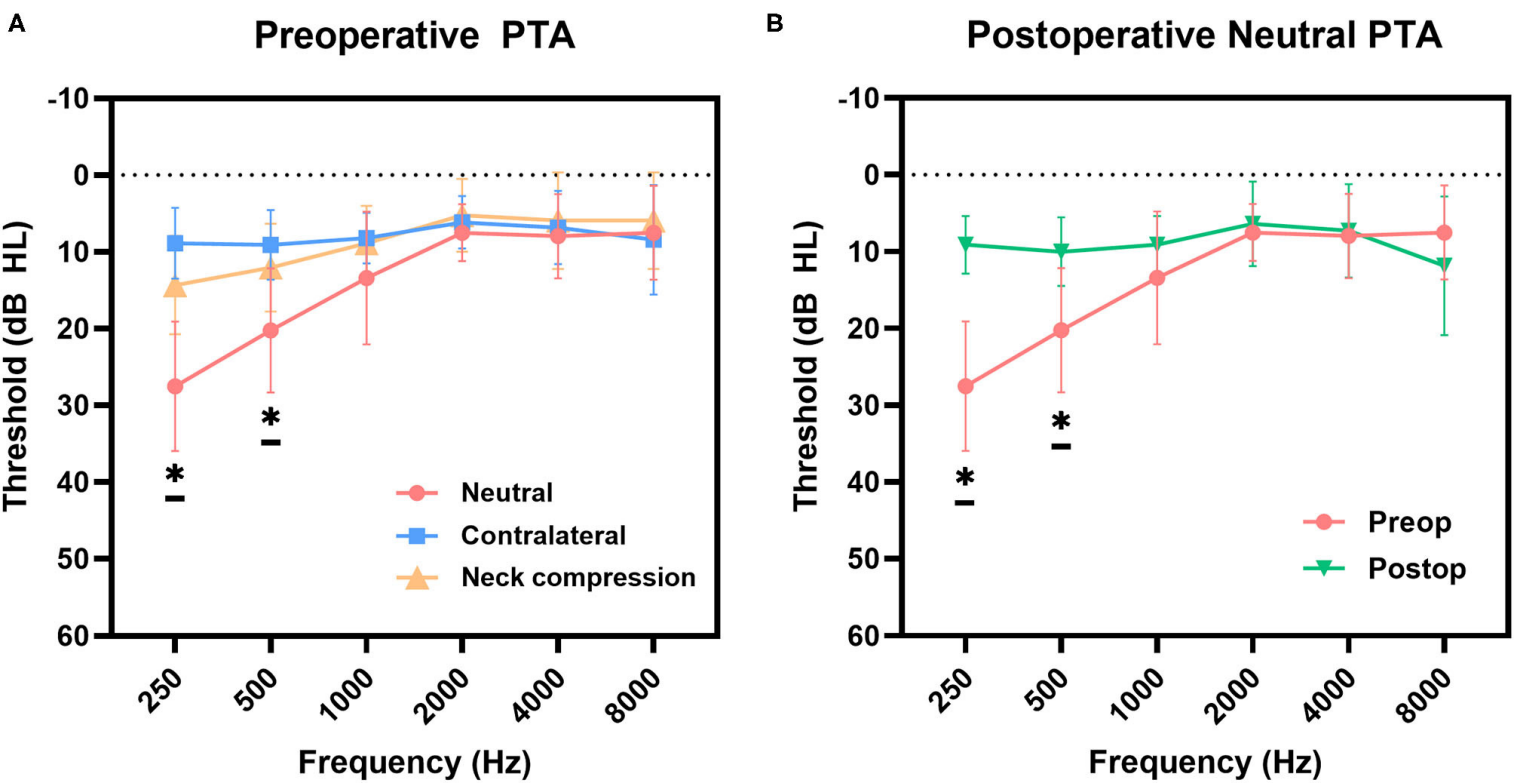
**(A)** Mean preoperative hearing thresholds of pure-tone audiogram (PTA) were compared pair-wise between the neutral and contralateral side, and between neutral and post-manual compression by Wilcoxon singed-rank test. Statistical significance results (*p* < 0.05) were obtained at 250 (*p* < 0.001) and 500 Hz (*p* < 0.001), respectively. **(B)** Average postoperative thresholds at 250 and 500 Hz significantly improved from those for the preoperative neutral position (*p* = 0.005 and *p* = 0.033, respectively, Wilcoxon signed-rank test), indicating successful treatment. The asterick “*” means that there is a statistical significance between Neutral and both Contralateral and Neck compression thresholds, between preop and postop thresholds.

Baseline and 3-month postoperative data for the audiological evaluations were available for 11 subjects. The postoperative average thresholds at 250 and 500 Hz (9.1 ± 3.6 dB HL and 10.0 ± 4.3 dB HL, respectively) significantly improved compared to the preoperative ipsilateral average pure-tone thresholds at the same frequencies (*p* = 0.005 and 0.033, the Wilcoxon signed rank test, respectively). The hearing thresholds at other frequencies (1,000, 2,000, 4,000, and 8,000 Hz) did not change postoperatively compared to the preoperative values. Figure 2 depicts the pre- and post-manual compression and postoperative PTA for a representative case. Importantly, the PTA thresholds at 250 and 500 Hz did not show any significant differences between preoperative audiometry parameters with ipsilesional neck compression compared to the postoperative ones (*p* = 0.053 and *p* = 0.603, respectively; Figure 3). Therefore, the average PTA improvements at 250 Hz and 500 Hz following manual neck compression indicated the degree of postoperative PTA improvements.

### Subjective Outcomes

**Figure 4:**
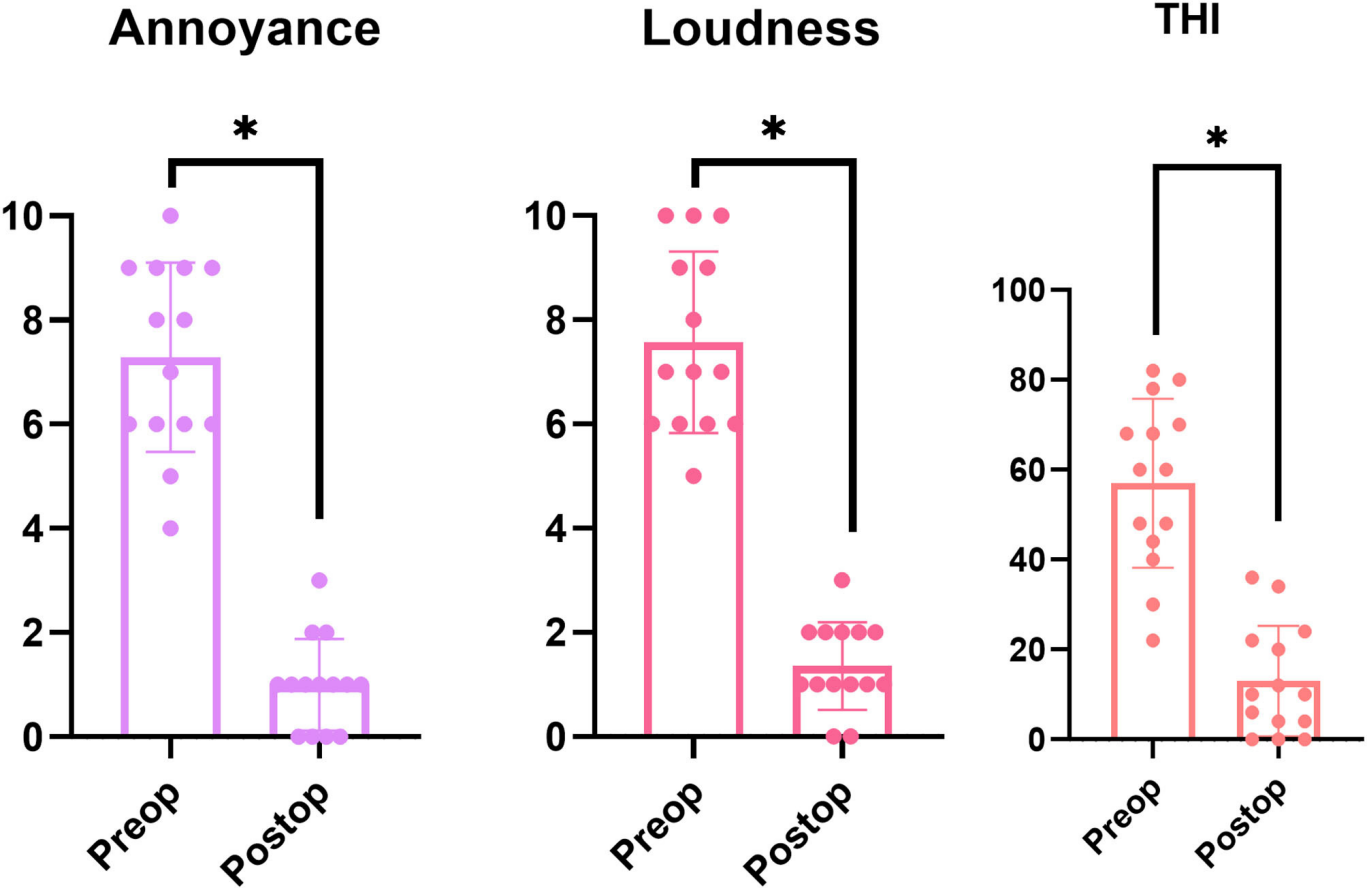
Pre- and post-operative Numerical Rating Scale (NRS) Annoyance and Loudness scales and Tinnitus Handicap Inventory (THI) scores. NRS Annoyance and Loudness scales significantly decreased after surgery in all 12 subjects (*p* = 0.001, Wilcoxon signed-rank test). With regard to THI scores, all 12 subjects reported significant improvement in postoperative tinnitus-related symptoms, with a statistically significant decrease from 54.1 ± 18.0 preoperatively to 14.3 ± 12.1 postoperatively (*p* = 0.002, Wilcoxon signed-rank test). NRS, numerical rating scale; THI, tinnitus handicap inventory. All 3 categories also showed statistical significance between preop and postop scores, which are also indicated with the asterick.

The median NRS-A significantly decreased from 7 (range, 4–10) to 1 (range, 0–3) (*p* = 0.001 by Wilcoxon signed-rank test), and the median NRS-L also significantly decreased from 7 (range, 5–10) to 1 (range, 0–6) (*p* = 0.001 by Wilcoxon signed-rank test) postoperatively (Figure 4). Furthermore, subjective PT improvements, as evidenced by change in median THI score, significantly improved from 60 (range, 22–82) preoperatively to 10 (range, 0–52) postoperatively (*p* = 0.002, Wilcoxon signed-rank test; Figure 4). Detailed scores of pre- and postoperative subjective outcomes are indicated in Table 3. In addition to written questionnaire results, all subjects answered that their symptoms had completely disappeared or much abated immediately and 3 months after the surgical interventions.

**Table 3:** Pre- and postoperative tinnitus questionnaire results.

	**Preoperative symptom scores**				**Postoperative symptom scores**			
**Pt. No**.	**NRS Annoyance**	**NRS Loudness**	**THI**		**NRS Annoyance**	**NRS Loudness**	**THI**	
1		N/A				N/A		
2	6	6	22	Mild	0	0	0	No
3	4	6	48	Moderate	3	1	24	Mild
4	6	6	40	Moderate	1	2	12	No
5	9	10	80	Severe	0	0	20	Mild
6		N/A				N/A		
7	6	6	30	Mild	1	1	0	No
8	5	7	44	Moderate	1	2	6	No
9	8	8	48	Moderate	1	1	10	No
10		N/A				N/A		
11	9	5	60	Severe	2	2	36	Mild
12	9	10	60	Severe	1	2	4	No
13	7	7	68	Severe	2	3	34	Mild
14	6	7	68	Severe	0	1	22	Mild
15	10	10	82	Severe	0	2	4	No
16		N/A				N/A		
17		N/A				N/A		
18		N/A				N/A		
19	9	9	78	Severe	1	1	0	No
20		N/A				N/A		
21		N/A				N/A		
22	8	9	70	Severe	1	1	10	No

*Pt., patient; No., number; NRS, numerical rating scale; THI, tinnitus handicap inventory; N/A, not available*.

*The median Numerical Rating Scale Annoyance and Loudness markedly decreased from 7 (range, 4–10) preoperatively to 1 (range, 0–3) postoperatively (p = 0.001) and from 7 (range, 5–10) preoperatively to 1 (range, 0–6) postoperatively (p = 0.001), respectively. Also, the median Tinnitus Handicap Inventory score significantly improved from 60 (range, 22–82) preoperatively to 10 (range, 0–52) postoperatively (p = 0.002)*.

### Negative Effect of Manual Neck Compression

Five out of 22 subjects (22.7%; Subject 5, 6, 7, 10, and 11) had negative manual compression effect (i.e., lack of improvement of ≥ 10 dB HL at 250 or 500 Hz) preoperatively, despite the presence of ipsilateral LFHL and radiologically-evident SS abnormalities. Only one subject (Subject 20) showed neither LFHL nor positive manual neck compression effect. Nonetheless, Subject 5, 7, and 11 underwent surgical interventions for each SS abnormality, which showed markedly improved postoperative PT symptoms with THI score (from 80 to 20, severe to mild; from 30 to 0, mild to none; from 60 to 36, severe to mild, respectively) and hearing thresholds at both 250 and 500 Hz. Subject 10 and 20 were not operated on due to mild PT symptoms. Their symptoms remained stable during the follow-up period. Although the PT symptom was severe in Subject 18 with both LFHL and positive neck compression effects, she did not want to undergo surgical treatment.

## Discussion

Since the first study by Sismanis proposed that digital pressure could be applied over the internal jugular vein to diagnose PT (19), many physicians have performed this examination to preoperatively characterize PT. The current study investigated the preoperative diagnostic and prognostic values of the effect of ipsilateral neck manual compression on the PT loudness and associated audiometric changes in subjects with SS-Deh and SS-Div. The new examination method, PTA with ipsilesional manual neck compression, may aid in having an initial diagnostic impression in SS-Div/SS-Deh-associated PT and identifying appropriate candidates for surgery.

Friedmann et al. suggested that cardiac venous pulsations from the dominant vein continuously collide with the inner venous walls and expand the dehiscent vascular portions, thereby increasing the diameter of the right-sided dominant vein (20). Furthermore, based on Poiseuille's law, the volumetric flow rate is mechanistically proportional to the radius to the fourth power. Variations in the SS geometry may accelerate the disruption of local laminar flow and cause continuous outward pressure, leading to SS-Deh and formation/enlargement of SS-Div (21). Therefore, turbulent blood flow due to hemodynamic changes, as well as vibration of a dehiscent vascular wall, even in the absence of turbulent flow, can transmit sounds into the cochlea, resulting in PT. Kao et al. discovered that distinct flow patterns (i.e., vortex pattern) within the internal jugular vein, which rely on the shape and position of the jugular bulbs, are closely associated with PT (22). The intensity of the vascular bruit is reflected by the venous flow velocity, which causes turbulence as it enters the SS. When the flow in the internal jugular vein is temporarily arrested due to external pressure (e.g., manual neck compression), venous flow velocity is minimized to an almost static condition, thereby eliminating the pulsatile perception (23). In the current study, the majority of subjects (16 subjects, 72.7%) showed positive neck compression effect and this phenomenon was consequently verified as the existence of venous origin of PT. Furthermore, our group again confirmed that SS resurfacing/reshaping, including external decompression or reduction of the SS, has produced satisfying long-term treatment outcomes.

Importantly, the combination of PTA and ipsileisional manual neck compression may help to confirm the venous origin of PT and may predict favorable surgical outcomes. In our previous study, subjects with PT and radiologically-evident vascular pathology near the cochlea frequently presented with ipsilateral LFHL (11, 12, 18). Previous studies have reported that intravascular flow produces PT, which is synchronous to the heartbeat. Additionally, the frequency spectrum of the first heart sound is primarily distributed in the low frequency range of 50–200 Hz (24). Specifically, in the cases of SS-Div/SS-Deh, it has been proven that pulse-synchronous acoustic characteristics are relatively low sound pressure levels within low-frequency range using TSR-STA (1). Therefore, PT may mask low-frequency sounds and result in LFHL. Our results are consistent with those of previous studies, which reported that the average PTA thresholds in the neutral position were significantly higher than those on the contralateral symptom-free side. In the present study, all but two subjects (Subjects 13 and 20) with ipsilateral LFHL had SS-Div/SS-Deh and the majority of the enrolled subjects had a positive manual compression effect in the affected ear. Furthermore, subjective PT improvements correlated with marked improvements in hearing thresholds at low frequencies after SS resurfacing/reshaping, similar to the threshold levels for the normal contralateral ear. These results suggest that the effect of ipsilesional manual neck compression on LFHL would be comparable to that of SS resurfacing/reshaping. Oh et al. reported that five patients with PT due to a dehiscent high jugular bulb demonstrated resolution of symptoms by ipsilateral internal jugular vein compression (25). In addition, Sismanis and Sismanis et al. reported resolution or improvement of PT by ipsilateral internal jugular vein compression, coupled with an improvement of LFHL, in patients with benign intracranial hypertension (19, 26). Expanding upon the improvement of LFHL in these previous studies, our study is meaningful in that the improvement in audiometric profiles can be visualized with the help of our new examination method and thus PTA with manual neck compression may aid in confirming a venous origin of PT and predicting a favorable surgical outcome.

This study had several limitations that should be addressed in future studies. First, the sample size was relatively small and only subjects with SS abnormalities were evaluated. A larger number of participants with various etiologies should be included in future studies. Second, this study only used TB-CTA or MRI/MRA to evaluate vascular abnormalities, which may have reduced the strength of evidence of alleviation of subjective symptoms. As previously mentioned, TSR-STA can also be used to evaluate the surgical outcomes (18, 27). Pre- and post-manual compression TSR-STA may provide an objective evaluation of PT changes, which will help clinicians identify patients who may benefit from surgery. Third, improvements in low-frequency hearing thresholds under manual compression showed wide variation. For instance, Subject 16 showed an improvement of 30 dB HL in the PTA thresholds at 250 Hz, while Subject 13 showed only an improvement of 5 dB HL at 250 Hz, despite applying manual compression. According to our hypothesis, if ipsilesional manual compression of the internal jugular vein is successful, the PTA threshold at low frequencies should improve up to the level of the normal contralateral ear because digital compression of the internal jugular vein limits the generation of PT. This variation among subjects may result from possible differences in the compression intensity among subjects. Therefore, future follow-up studies should minimize these errors by controlling the intensity of compression (i.e., to the level of disappearance or stark alleviation of PT). Fourth, evaluation of the frequency of PT using 250 and 500 Hz can be somewhat less comprehensive since spectro-temporal analyses may present pulse-synchronous fluctuation of tinnitus frequency above 500 Hz occasionally. Thus, evaluating additional low frequencies (e.g., 750 Hz) will be helpful to better understand the effect of manual neck compression on pulse-synchronous acoustic characteristics of PT in the future studies. Nevertheless, we believe that our findings of convenient and cost-effective use of PTA with manual neck compression are clinically meaningful, especially when the PTA shows ipsilateral LFHL in PT patients.

## Conclusion

This was the first study to explore the preoperative significance of ipsilateral manual neck compression in patients with pulsatile tinnitus secondary to SS-Deh/Div. A positive manual compression effect in the affected ear strongly suggested postoperative improvements in ipsilateral LFHL and PT symptoms. Temporary improvement in PT and LFHL with manual compression over the ipsilateral neck may help to confirm the venous origin of the PT and predict a favorable surgical outcome following repair of SS-Deh or SS-Div.

## Data Availability Statement

The original contributions presented in the study are included in the article/supplementary material, further inquiries can be directed to the corresponding author.

## Ethics Statement

The studies involving human participants were reviewed and approved by Institutional Review Board of the Clinical Research Institute (no. B-2107-699-105). The patients/participants provided their written informed consent to participate in this study. Written informed consent was obtained from the individual(s) for the publication of any potentially identifiable images or data included in this article.

## Author Contributions

J-JS designed the study. SL led the analysis, interpretation of the results, and drafted the first manuscript. S-YL, BC, J-WK, and SH reviewed and revised the manuscript for intellectual contents. SL, S-YL, BC, J-WK, SH, and J-JS contributed to all aspects of the investigation, including data collection, analysis, interpretation of the results, and revision of the manuscript for important intellectual contents. All authors approved the final version of the manuscript and agree to be accountable for all aspects of the work.

## Funding

This work was supported by grants from the National Research Foundation of Korea funded by the Korean government (MSIP) (no. NRF-2022R1A2B5B02002139; recipient: J-JS).

## Conflict of Interest

The authors declare that the research was conducted in the absence of any commercial or financial relationships that could be construed as a potential conflict of interest.

## Publisher's Note

All claims expressed in this article are solely those of the authors and do not necessarily represent those of their affiliated organizations, or those of the publisher, the editors and the reviewers. Any product that may be evaluated in this article, or claim that may be made by its manufacturer, is not guaranteed or endorsed by the publisher.

## References

[1] Song JJ An GS Choi I De Ridder D Kim SY Choi HS. Objectification and differential diagnosis of vascular pulsatile tinnitus by transcanal sound recording and spectrotemporal analysis: a preliminary study. Otol Neurotol. (2016) 37:613–20. 10.1097/MAO.0000000000001005 27023015

[2] Song JJ De Ridder D Schlee W Van De Heyning P Vanneste S. “Distressed aging”: the differences in brain activity between early- and late-onset tinnitus. Neurobiol Aging. (2013) 34:1853–63. 10.1016/j.neurobiolaging.2013.01.014 23415838

[3] Lee SY Choi BY Koo JW De Ridder D Song JJ. Cortical oscillatory signatures reveal the prerequisites for tinnitus perception: a comparison of subjects with sudden sensorineural hearing loss with and without tinnitus. Front Neurosci. (2020) 14:596647. 10.3389/fnins.2020.596647 33328868 PMC7731637

[4] Sismanis A. Pulsatile tinnitus: contemporary assessment and management. Curr Opin Otolaryngol Head Neck Surg. (2011) 19:348–57. 10.1097/MOO.0b013e3283493fd8 22552697

[5] Baguley D Mcferran D Hall D. Tinnitus. Lancet. (2013) 382:1600–7. 10.1016/S0140-6736(13)60142-7 23827090

[6] Liyanage SH Singh A Savundra P Kalan A. Pulsatile tinnitus. J Laryngol Otol. (2006) 120:93–7. 10.1017/S0022215105001714 16359136

[7] Ahsan SF Seidman M Yaremchuk K. What is the best imaging modality in evaluating patients with unilateral pulsatile tinnitus? Laryngoscope. (2015) 125:284–5. 10.1002/lary.24822 25042105

[8] Carlson ML Sweeney AD Pelosi S Wanna GB Glasscock ME 3rd Haynes DS. Glomus tympanicum: a review of 115 cases over 4 decades. Otolaryngol Head Neck Surg. (2015) 152:136–142. 10.1177/0194599814555849 25385810

[9] Grewal AK Kim HY Comstock RH 3rd Berkowitz F Kim HJ Jay AK. Clinical presentation and imaging findings in patients with pulsatile tinnitus and sigmoid sinus diverticulum/dehiscence. Otol Neurotol. (2014) 35:16–21. 10.1097/MAO.0b013e31829ab6d7 24005164

[10] Eisenman DJ Raghavan P Hertzano R Morales R. Evaluation and treatment of pulsatile tinnitus associated with sigmoid sinus wall anomalies. Laryngoscope. (2018) 128(Suppl. 2):S1–13. 10.1002/lary.27218 29756346

[11] Lee SY Kim MK Bae YJ An GS Lee K Choi BY. Longitudinal analysis of surgical outcome in subjects with pulsatile tinnitus originating from the sigmoid sinus. Sci Rep. (2020) 10:18194. 10.1038/s41598-020-75348-3 33097817 PMC7584625

[12] Lee SJ Lee SY An GS Lee K Choi BY Koo JW. Treatment outcomes of patients with glomus tympanicum tumors presenting with pulsatile tinnitus. J Clin Med. (2021) 10:2348. 10.3390/jcm10112348 34071897 PMC8198089

[13] Jeon HW Kim SY Choi BS Bae YJ Koo JW Song JJ. Pseudo-low frequency hearing loss and its improvement after treatment may be objective signs of significant vascular pathology in patients with pulsatile tinnitus. Otol Neurotol. (2016) 37:1344–9. 10.1097/MAO.0000000000001179 27525714

[14] Sismanis A Smoker WR. Pulsatile tinnitus: recent advances in diagnosis. Laryngoscope. (1994) 104:681–8. 10.1288/00005537-199406000-00007 8196443

[15] Bhatnagar K Lataille AT Eisenman DJ. Patterns of audiometric threshold shifts from pulsatile tinnitus due to sigmoid sinus wall anomalies. Am J Otolaryngol. (2020) 41:102647. 10.1016/j.amjoto.2020.102647 32683189

[16] Newman CW Jacobson GP Spitzer JB. Development of the tinnitus handicap inventory. Arch Otolaryngol Head Neck Surg. (1996) 122:143–8. 10.1001/archotol.1996.01890140029007 8630207

[17] Song JJ Kim YJ Kim SY An YS Kim K Lee SY. Sinus wall resurfacing for patients with temporal bone venous sinus diverticulum and ipsilateral pulsatile tinnitus. Neurosurgery. (2015) 77:709–17. discussion 717. 10.1227/NEU.0000000000000902 26197352

[18] Kim SH An GS Choi I Koo JW Lee K Song JJ. Pre-treatment objective diagnosis and post-treatment outcome evaluation in patients with vascular pulsatile tinnitus using transcanal recording and spectro-temporal analysis. PLoS ONE. (2016) 11:e0157722. 10.1371/journal.pone.0157722 27351198 PMC4924851

[19] Sismanis A. Otologic manifestations of benign intracranial hypertension syndrome: diagnosis and management. Laryngoscope. (1987) 97:1–17. 10.1288/00005537-198708001-00001 3302575

[20] Friedmann DR Eubig J Mcgill M Babb JS Pramanik BK Lalwani AK. Development of the jugular bulb: a radiologic study. Otol Neurotol. (2011) 32:1389–95. 10.1097/MAO.0b013e31822e5b8d 21921860

[21] Sutera SP Skalak R. The history of Poiseuille's law. Annu Rev Fluid Mech. (1993) 25:1–20. 10.1146/annurev.fl.25.010193.000245

[22] Kao E Kefayati S Amans MR Faraji F Ballweber M Halbach V. Flow patterns in the jugular veins of pulsatile tinnitus patients. J Biomech. (2017) 52:61–7. 10.1016/j.jbiomech.2016.12.008 28057349 PMC5415495

[23] Berguer R Nowak P. Treatment of venous pulsatile tinnitus in younger women. Ann Vasc Surg. (2015) 29:650–3. 10.1016/j.avsg.2014.12.039 25752987

[24] Stein PD Sabbah HN Lakier JB Magilligan DJ Jr Goldstein D. Frequency of the first heart sound in the assessment of stiffening of mitral bioprosthetic valves. Circulation. (1981) 63:200–3. 10.1161/01.CIR.63.1.200 7438394

[25] Oh SJ Kim D Lee JI Ko JK Choi SW Kong SK. Transvenous stent-assisted coil embolization for management of dehiscent high jugular bulb with tinnitus and contralateral hypoplastic venous sinuses. Otol Neurotol. (2019) 40:1253–9. 10.1097/MAO.0000000000002349 31469796

[26] Sismanis A Butts FM Hughes GB. Objective tinnitus in benign intracranial hypertension: an update. Laryngoscope. (1990) 100:33–6. 10.1288/00005537-199001000-00008 2293699

[27] Lee SY Song SK Park SJ Park HG Choi BY Koo JW. Jugular bulb resurfacing with bone cement for patients with high dehiscent jugular bulb and ipsilateral pulsatile tinnitus. Otol Neurotol. (2019) 40:192–9. 10.1097/MAO.0000000000002093 30624401

